# A Novel Therapy for Melanoma Developed in Mice: Transformation of Melanoma into Dendritic Cells with *Listeria monocytogenes*

**DOI:** 10.1371/journal.pone.0117923

**Published:** 2015-03-11

**Authors:** Lucia Bronchalo-Vicente, Estela Rodriguez-Del Rio, Javier Freire, Ricardo Calderon-Gonzalez, Elisabet Frande-Cabanes, Jose Javier Gomez-Roman, Hector Fernández-Llaca, Sonsoles Yañez-Diaz, Carmen Alvarez-Dominguez

**Affiliations:** 1 Grupo de Genómica, Proteómica y Vacunas, Instituto de Investigación Marqués de Valdecilla (IDIVAL), Santander, Spain; 2 Servicio de Dermatología, Hospital Universitario Marqués de Valdecilla, Santander, Cantabria, Spain; 3 Servicio de Anatomía Patológica, Hospital Universitario Marqués de Valdecilla, Santander, Cantabria, Spain; Indian Institute of Science, INDIA

## Abstract

*Listeria monocytogenes* is a gram-positive bacteria and human pathogen widely used in cancer immunotherapy because of its capacity to induce a specific cytotoxic T cell response in tumours. This bacterial pathogen strongly induces innate and specific immunity with the potential to overcome tumour induced tolerance and weak immunogenicity. Here, we propose a *Listeria* based vaccination for melanoma based in its tropism for these tumour cells and its ability to transform *in vitro* and *in vivo* melanoma cells into matured and activated dendritic cells with competent microbicidal and antigen processing abilities. This *Listeria* based vaccination using low doses of the pathogen caused melanoma regression by apoptosis as well as bacterial clearance. Vaccination efficacy is LLO dependent and implies the reduction of LLO-specific CD4^+^ T cell responses, strong stimulation of innate pro-inflammatory immune cells and a prevalence of LLO-specific CD8^+^ T cells involved in tumour regression and *Listeria* elimination. These results support the use of low doses of pathogenic *Listeria* as safe melanoma therapeutic vaccines that do not require antibiotics for bacterial removal.

## Introduction

Coley reported for the first time cancer therapies based on the use of bacteria after observation of the tumour regression in a patient after bacterial infection [1]. During the early development of bacterial-based anticancer therapies, different anaerobic bacteria were used that can proliferate in a hypoxic environment such as that of tumours. Some examples are *Clostridium novyi*, *Bifidobacteria*, *Streptococcus pyogenes* or *Vibrio cholera*. The use of bacteria as anticancer agents was followed by antibiotic treatment to control the infection. However, current bacterial-based anticancer therapies use attenuated strains of *Salmonella* and *Listeria monocytogenes* (LM) expressing specific antigens to target them to tumours and reduce tumour burden [2,3].

*Listeria monocytogenes* based vaccine vectors with attenuated strains show high efficiency against several tumours such as neuroglioma, hepatocellular carcinoma, cervical, prostate, and breast cancers, with a bacterial burden 500-fold higher than the classical doses used with pathogenic LM^WT^ [4,5]. The efficiency of *Listeria* based vaccines depends on the ability of the bacteria to induce a strong innate and T-cell based immunity playing an important role CD11c^+^ dendritic cells (DCs) together with the pathogen’s capacity to overcome tolerance to self-antigens such as tumour antigens [6].

Melanomas are malignant forms of melanocytes and common skin cancers, with an incidence of 2%. They are particularly aggressive in highly photosensitive skin types. In our institution, the rate of incidence of melanoma increased by 3% in the last 2 years, becoming the most prevalent cancer among young women under the age of 40 years [7]. Melanoma is a low immunogenic tumour characterized by the aberrant expression of MHC class II molecules that might present melanoma-associated antigens to specific CD4^+^ T cells [8]. However, melanoma MHC class II expression correlates with metastatic disease and poor survival [9] and the melanoma specific CD4^+^ T cells appeared as tumour tolerogenic. Murine melanoma models that mimic the aggressive behaviour of metastatic melanoma, such as B16F10 cells, have been developed, and similar to humans, they also express MHC class II molecules [10].

Bacterial infection in melanoma or melanocytes has not been investigated in detail, and only rare infections have been reported such as non-tuberculosis *Mycobacterium* infection or abscesses of *Salmonella enterica* at the site of melanoma metastasis [11–13]. Several cases of cutaneous listeriosis in adults have also been reported; suggesting that infection of melanocytes with *Listeria* is possible [14], although LM infection of melanoma or melanocytes is not well understood. Furthermore, most cases of neonatal listeriosis show diffuse skin lesions. Here, we hypothesized that *Listeria* tropism for melanoma might cause activation of the anti-melanoma immune response, blocking tumour progression. In the present study, we test this hypothesis performing a detailed analysis of the phagocytic abilities of melanoma cells after *Listeria* infection and propose a *Listeria* based vaccination for experimental melanoma.

## Materials and Methods

### Cells, recombinant proteins and peptides

Murine melanoma B16F10 cells were a gift from B. Alarcon and acquired from ATCC, human melanoma cell lines A-375 and Mel-H0, were a gift from M.D Boyano-Lopez and acquired from ATCC, J-774 murine macrophages and CHO cells were acquired from ATCC. Bone-marrow-derived dendritic cells (BMDC) were obtained from femurs of C57BL/6, Balb/c or CD-1 mice differentiated with 25 ng/ml granulocyte-macrophage colony-stimulating factor (GM-CSF) for 7 days as described [15]. Murine embryonic fibroblasts were cultured after thawing as previously described [16]. On day 7, we performed a positive selection of CD11c^+^-BMDC cells using magnetic beads and MACS separation columns (Miltenyi Biotech Inc., Auburn, CA) [15]. *E*. *coli* strain BL21 expressing large quantities of His-fusion recombinant LLO protein (LLO_rec_) was provided by *D*.*A*. *Portnoy*. *F*. *Roncal* synthesized LLO_296–304_ and LLO_190–201_ peptides (CNB, CSIC, Madrid) followed by HPLC and Mass Spectrometry using a MALDI-TOF Reflex IV mass spectrometer (Bruker Daltonics, Bremen, Germany). Peptide purity was > 95% after HPLC. B16F10 cells (2 x 10^9^) were homogenized as previously described for J-774 cells and precipitated with TCA and washed with PBS to obtain a B16F10 extract (MEL_ext_) (1 mg/ml).

### Bacteria. *L*. *monocytogenes*

10403S strain (LM^WT^) and the *hly*-deficient LM mutant (LM^ΔLLO^, DPL-2161 strain) were obtained from D.A. Portnoy (University of California, Berkeley, CA, USA) and green fluorescent protein (GFP)–LM variant of LM^WT^ strain DH-L1039 (GFP-LM^WT^) from D.E. Higgins (Harvard Medical School, Boston, MA, USA).

### Kinetic infection assays

CD11c^+^-BMDC, A-375, Mel-H0 and B16F10 cells were infected with LM^WT^ or LM^ΔLLO^ at a ratio of 10:1 (bacteria: cells) for different times (0, 4, 8 or 16 h) as described [15]. J-774, MEF and CHO cells were infected as previously reported [16]. Colony forming units (CFU) were examined in blood agar plates and results are the mean ± SD.

### Replication indexes of LM strains in melanoma and BMDC

Replication indexes (RIs) were calculated in CD11c^+^-BMDC, A-375, Mel-H0 and B16F10 cells infected as ratios of CFU at 16 h to CFU at 0 h ± SD of triplicates as reported [16, 17]. RI values higher than 25, represent a rapid LM proliferation and poor bactericidal abilities of the cells. Phagocytic rates corresponded to bacteria internalization rates at 0 h.

### Phagosome isolation and cytosolic fractions

CD11c^+^-BMDC, A-375, Mel-H0 and B16F10 cells were infected for 1 hour and homogenized in HBE buffer (250 mM sucrose, 0.5 mM EGTA, 20 mM HEPES-KOH, pH 7.2) to obtain post-nuclear supernatants (PNS). Phagosomes were isolated from an aliquot of PNS into a 20% sucrose gradient as described [16, 17]. An additional PNS was used to estimate the percentage of total internalized bacteria expressed as CFU. The percentages of phagosomal and cytosolic bacteria were calculated according to the following ratio, PNS—phagosomal numbers/PNS × 100, as described [16, 17].

### Phagosome western blotting and immunoprecipitations

Phagosomes isolated from BMDCs, B16F10, J-774, MEF or CHO cells infected with LM^WT^ for 20 min, were solubilized and CFU analysed in blood agar plates. MHC-II and Rab5a molecules were detected by western blotting and the two forms of LLO bound to MHC class II molecules, LLO_intact_ and processed LLO_1–491_ forms were detected after immunoprecipitation with anti-MHC-II antibodies followed by western-blot with rabbit anti-LLO antibody as reported [17].

### Confocal microscopy

B16F10 and BMDC cells were infected with GFP-LM for 1 h, washed, fixed in 3% paraformaldehyde and permeabilized with PBS-0.05% Triton X-100 as reported [15–17]. Confocal microscopy imaging was performed with a Nikon A1R confocal microscope.

### B16F10 induction of carcinomatous peritonitis and LM vaccination

C57BL/6 female mice (n = 5) were inoculated in the peritoneal cavity (*i*.*p*) with 5 × 10^5^ B16F10 cells for 7, 15 or 23 days. For LM^WT^ vaccinations, mice inoculated *i*.*p* with B16F10 for 7 days were next infected *i*.*p* with 5 × 10^3^ GFP-LM (CFU/mice) (n = 5) for 5 days to analyse LM innate and specific immunity. Mice were bled before sacrifice and sera stored at −80°C to measure cytokines by FACS analysis. Spleens, peritoneal melanoma and lungs were recovered and photographed. Spleens and melanoma were further processed for FACS analysis and an aliquot plated into blood-agar plates to count CFU.

### Immunohistochemistry

Mice were sacrificed on days 7, 15 and 23 after melanoma inoculation. The most common metastatic organs (liver, spleen, kidney, adrenal glands, liver and lungs) were resected, sectioned, and fixed by immersion in 4% formaldehyde for 24 h. Organs were subsequently embedded in paraffin and cut at 3-μm thickness for histological analysis. Different sections (stained with hematoxylin-eosin) of each organ were analysed by two independent pathologists. For immunohistochemical analysis of lymphocyte markers, EnVision technology (Dako) was used. Samples were boiled in target retrieval solution buffer (pH 6 for CD23, pH 8 for the rest) for 20 min, subsequently cooled in distilled water and 1×PBS. Next, the sections were incubated with ready-to-use primary monoclonal antibodies (Dako) against CD4 (clone 4B12), CD8 (clone c8/144B), CD23 (clone DAK-CD23), CD45 (clone 2B11 + PD7/26), CD56 (clone 123C3) and CD68 (clone KP1). The antigens were visualized using biotinylated antibodies and streptavidin conjugated with horseradish peroxidase (EnVision Mouse HRP, Dako). Diaminobenzidine (DAB, Dako) was used as the chromogen. Antigen concentrations were all determined based on the percentage and intensity of tumour cells showing positive staining.

### FACS analysis of spleens, melanoma, intracellular IFN-gamma staining and cytokine measurements

Cell surface markers of J-774 macrophages, BMDCs or B16F10 melanoma infected or not with different LM strains (LM^WT^, GFP-LM^WT^ or LM^ΔLLO^) or spleens and recovered melanoma from mice treated with B16F10 and infected with the above mentioned LM strains were analysed by FACS for the following markers: CD4-PE, CD8α-PE, CD49b-PE, F4/80-PE, CD11b-APC, CD11c-PE, MHC-II-APC, CD40-PE, CD83-APC, CD86-V450 and iNOS/type II-FITC. Melanoma cells from mice were also analysed for apoptosis by FACS analysis using Annexin-V-APC and 7-AAD (BD-Biosciences). Mice sera or supernatants of B16F10 cells infected or not with LM^WT^ for 24 hours were used to quantify cytokines using the CBA kit (Becton Dickinson, Palo Alto, CA, USA). Samples were analysed in triplicate and results were expressed as the mean ± SD of two separate experiments. For measuring of intracellular IFN-γ, spleen cells were cultured in 96-well plates (5 x 10^6^ cells/ml) and stimulated with recombinant LLO (0.1 μg/ml), B16F10 extract (50 μg/ml) (MEL_ext_), LLO_190–201_ (50 μM) or LLO_296–304_ peptides (50 μM) for 5 h in the presence of brefeldin A [15, 17]. Next, cells were surface labelled for CD4 or CD8, fixed and permeabilized with cytofix/cytoperm kit to measure intracellular IFN-gamma (BD Biosciences). After sample acquisition, data were gated for CD4^+^ or CD8^+^ events, and the percentages of these cells expressing IFN-gamma were determined according with the manufacturer’s recommendations. Results were corrected according to the percentages of total CD4^+^ or CD8^+^ positive cells. Data were analysed using FlowJo software (Treetar, Ashland, OR).

### Frequencies of LLO-peptide specific CD8^+^ T cells

To confirm the frequency of LLO_296–3049_-specific CD8 T cells producing IFN-gamma, we used recombinant soluble dimeric mouse H-2K^b^:Ig fusion protein following the instructions of the manufacturer (DimerX I; BD Bioscience). LLO_296–304_ peptide (40 μM) was pre-incubated with PE-conjugated H-2K^b^:Ig (1 μM) in PBS, at 37°C for 16 h as previously described [15]. Splenocytes (2 × 10^7^ cells/ml) were incubated with IFN-gamma and CD8 antibodies and the staining cocktail mix described above for 10 min at 4°C. Percentages of CD8^+^ gated cells were expressed as the mean ± SD of triplicates (P < 0.05). Data were analysed using FlowJo software.

### Measurement of NO production

B16F10 and BM-DC were infected with LM or not and assayed for NO production as previously reported [18].

### B16F10 subcutaneous tumour model and LM vaccination

Newborn C57BL/6, Balb/c or CD-1 mice of 2 days old (n = 10) were inoculated in the back subcutaneously (*s*.*c*) with 1 x 10^5^ B16F10 cells for 7 days to develop a measurable (4–6 mm) tumour. For LM^WT^ vaccinations, mice inoculated *s*.*c* with B16F10 for 7 days were next infected *s*.*c* with 1 x10^3^ LM^WT^ (CFU/mice) (n = 10) for 5 days. Mice were sacrificed and tumours weighted and sizes measured with a calliper and photographed to obtain images. Values shown for tumour size (mm) were the mean ± SD of three different experiments.

### Statistical analysis

Student’s *t* test was used for statistical analysis and analysis of variance (ANOVA) for cytokine measurements. Data were analysed using FlowJo software (Treestar, Ashland, OR). *P* ≤ 0.05 was considered significant. GraphPad software was used for graphic presentation.

### Ethics statement

This study was carried out in strict accordance with the recommendations in the Guide for the Care and Use of Laboratory Animals of the Spanish Ministry of Science, Research and Innovation. The Committee on the Ethics of Animal Experiments of the University of Cantabria approved this protocol (Permit Number: 2012/06) that follows the Spanish legislation (RD 1201/2005). All surgery was performed under sodium pentobarbital anaesthesia, and all efforts were made to minimize suffering.

## Results and Discussion

Coley’s observations that a neck cancer patient recovered after infection with erysipelas initiated the use of bacteria and their toxins to treat end stage cancers [3]. However, pathogenicity and toxicity are important concerns limiting the broad clinical application of bacteria as anticancer agents. In this regard, the adjuvant features of LM involve its ability to target and activate DCs and its dual intracellular distribution in phagosomes and cytosol, which allows the activation of LM specific CD4^+^ and CD8^+^ T cells. However, two additional LM characteristics seemed also relevant to target tumour cells [5] and to live under hypoxic conditions. Attenuated LM strains on a truncated form of listeriolysin O (LLO) fused to tumour antigens appeared as promising vaccines for cervical and breast cancer [4,5]. These cancer vaccines exerted their effect through out the killing of tumour cells by CD8^+^ cytotoxic responses to tumour antigens. However, there are other mechanisms by which *Listeria*-based cancer vaccines show their actions, independent of tumour antigens. Here, we present a dramatic effect of LM^WT^ vaccination on an experimental melanoma model with poor immunogenicity, the B16F10 induction of cancerous peritonitis. We provide evidence of melanoma growth reduction after LM^WT^ vaccination and transformation of tumour cells into activated dendritic cell phenotypes that activates *Listeria*-specific CD8 T cell responses and innate pro-inflammatory responses that in turns eliminated the pathogen.

### *In vitro Listeria* induced transformation of melanoma into dendritic cells

Melanoma is one of the fastest growing cancers in the world, and surgery is the best available therapy for this malignancy both during the early developmental stages and in advanced metastatic melanoma. Therefore, alternative measures are needed as the one proposed in this study using low doses of pathogenic LM^WT^ as a vaccination therapy. This therapy is based in the high tropism of LM for melanoma [5,19] and the transformation of these tumour cells into dendritic-like cell phenotypes functioning as competent APC.

**Fig 1:**
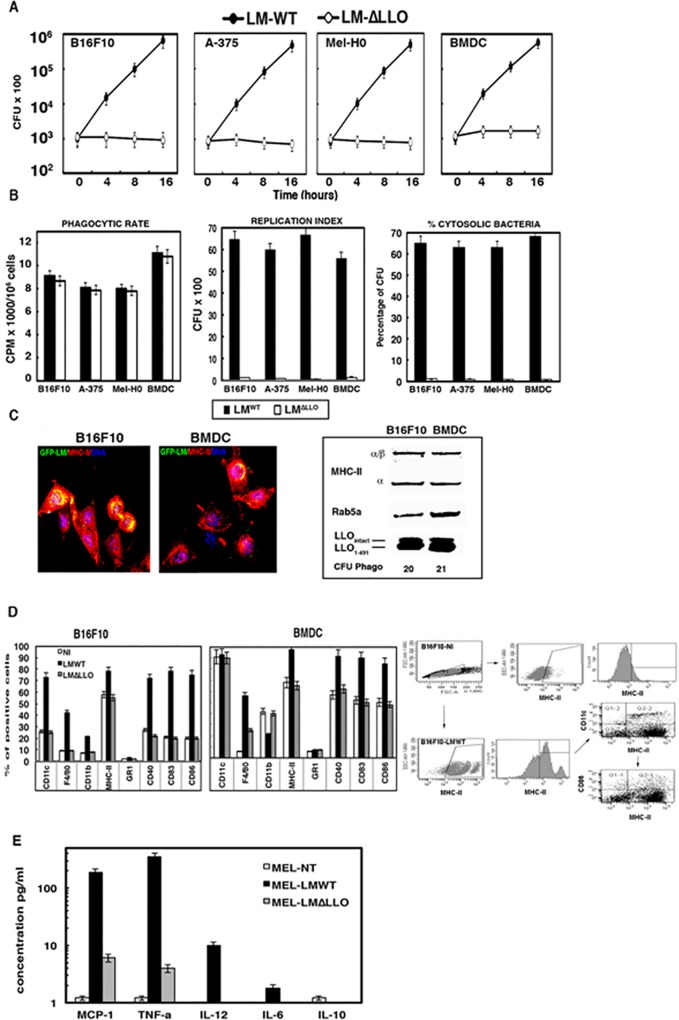
*Listeria* induced transformation of melanoma into dendritic cells. ***A***, Kinetic analysis of BMDC, murine (B16F10) and human (A-375 and Mel-H0) melanoma cells infected with different LM strains (LM^WT^, LM^ΔLLO^). Results are expressed as CFU (mean ± SD) obtained with triplicate samples from three independent experiments (*P*<0.05). ***B***, Different phagocytic parameters analysed in melanoma and BMDC: phagocytic rates after incubation with [^35^S]-labelled LM strains for 45 min (left plot). Radioactivity associated with cell lysates (CPM) was quantified in a β2 counter as the bacterial phagocytic rates. Results are expressed as cpm of internalized bacteria (mean ± SD) (*p* < 0.05). Replication indexes (RI) analysis is shown in middle plot. RIs were calculated as the ratio of the number of CFU at 16 h divided by the amount of CFU at 0 h. This parameter was considered as an indicator of bacterial growth. Results are expressed as CFU (mean ± SD) (*p* < 0.05). The percentages of cytosolic fractions are shown in right plot after purification of phagosomal and cytosolic fractions as in *Material and Methods*. Results are expressed as percentages of total internalized CFU in PNS (mean ± SD) (*p* < 0.05). ***C***, Images correspond to confocal microscopy examination of melanoma and BMDC infected with GFP-LM^WT^. GFP-LM^WT^ (green channel) co-localize with MHC-II molecules (red channel). Western blots correspond to the analysis in purified phagosomes for different MIIC markers: a/b stable MHC-II chains; Rab5a and LLO forms bound to MHC-class II molecules. CFU values of purified phagosomes are shown below western blots. ***D***, BMDC and B16F10 infected with LM strains or non-infected (NI) were surface stained for the following markers: CD11c-PE, CD11b-FITC, F4/80-PE, CD40-PE, Gr-1-FITC and anti-MHC-II-APC. Samples were acquired using FACSCanto flow cytometer and percentages of positive cells for each antibody are shown. Results are expressed as the mean ± SD of triplicates (*p*<0.05). ***E***, Same melanoma cells infected with different LM strains or non-infected (NI) as in D for 24 hours. Supernatants were recovered, filtered through 3 μm syringe to discard bacteria and the levels of pro-inflammatory cytokines MCP-1, TNF-alfa, IL-6, IL-10 or IL-12 were analysed using the CBA kit (Becton Dickinson) by flow cytometry. Results were expressed as cytokine concentration (pg/ml of mean ± SD, *P*<0,05).

We first observed that murine or human melanoma cell lines infected with pathogenic LM^WT^, transformed these malignant melanocytes into professional antigen presenting cells, APC, with a phenotype and function analogous to those of skin DCs, also known as Langerhans cells. In this regard, LM^WT^ murine showed a characteristic exponential proliferation in murine (B16F10) or human melanoma cell lines (A-375 or Mel-H0) (black circles in Fig. 1A) and high phagocytic rates (left plot of Fig. 1B). The LM *hly*-deficient strain, LM^ΔLLO^, which has a gene deletion that renders it unable to escape from phagosomes, did not replicate in any melanoma cell line (open circle plots of Fig. 1A) and suggested that melanoma posed listericidal capacity. In fact, pathogenic LM^WT^ presented high replication indexes, RI, (RI > 25) in melanoma and BMDC and the majority of the bacteria localized in the cytosol, 65–68% of LM^WT^ (middle and right plots of Fig. 1B). APC such as BMDC or melanoma showed medium ranges of listericidal abilities since LM^ΔLLO^ mutants showed no intracellular growth (Fig. 1A) [15], low RI values (RI < 1) (white bars in middle plot of Fig. 1B) and almost no bacteria localized in the cytosol (white bars in right plot of Fig. 1B). However, J-774 macrophages are APC with higher listericidal abilities since LM^ΔLLO^ mutants become degraded (RI < 0.1) (*S1A Fig.*). On the other side, non-APC cell types such as mouse embryonic fibroblasts (MEF) or the ovarian CHO tumour cells lacked listericidal abilities since both LM^WT^ and LM^ΔLLO^ showed exponential intracellular growth as previously reported [16] (*S1A Fig.*). These results strongly suggested that melanoma function as professional phagocytes. Next, we verified APC function examining the antigen processing compartments (MIIC) competency. Melanoma cells infected with LM^WT^, showed phagosomal-like structures with significant co-localization of green-fluorescent bacteria, GFP-LM^WT^ and MHC-II molecules (yellow fluorescence in Fig. 1C), reflecting high MIIC competency. Similarly, purified phagosomes of melanoma and BMDC presented high levels of classical MIIC markers [16, 17] such as the endosomal-phagosomal regulator Rab5a, stable a/b MHC-II molecules and significant amounts of LLO_1–491_ processed forms that bound to MHC class II molecules (western blots in Fig. 1C and *S1B Fig.*). They also presented lysosomal enzymes involved in LLO processing such as cathepsin-D (*S1B Fig.*) and low CFU values confirming their microbicidal capacities (CFU Phago values below western blots of Fig. 1C) [16–18]. J-774 macrophages presented almost all MIIC markers except for low levels of cathepsin-D, while non-APC cells as MEF or CHO exclusively showed the MIIC marker Rab5a, indicating very low MIIC competency (*S1B Fig.*) [15, 18].

Since the characteristic phenotype of B16F10 melanoma, namely MHC-II^+^CD11c^+/-^CD40^+/-^CD83^+/-^CD86^+/-^CD11b^-^CD8α^-^F4/80^-^Gr-1^-^ (white bars in the right plot of Fig. 1D) [20], also resembles that of immature DCs [21], we next evaluated LM^WT^ effect on cell surface markers. LM^WT^ transformation of melanoma resulted in a shift to a phenotype characteristic of matured DCs, namely MHC-II^++^CD11c^++^CD40^+^CD83^+^CD86^+^CD11b^-^CD8α^+^iNOS^+^F4/80^-^Gr-1^-^ (black bars in Fig. 1D and Table 1). This melanoma transformation required LLO since LM^ΔLLO^ mutants caused no change in the phenotype (grey bars in Fig. 1D). The strategy was as follows; first we selected on the FACS plots of non-infected melanoma (B16F10-NI in Fig. 1D-FACS plots on right), the single positive MHC-II cells. Using same parameters, we selected on FACS plots of melanoma-infected cells (B16F10-LMWT in Fig. 1D-FACS plots on the right), the single positive MHC-II cells. Next, we selected the double positive MHC-II and CD11c or CD40 or CD83 or CD86 cells (last arrow sequence in B16F10-LMWT plots in Fig. 1D-FCAS plots on the right). Infection of human melanoma A375 and Mel-H0 with LM^WT^ also shifted the phenotype towards an activated DC-like phenotype characterized by HLA-DR^+^CD11c^+^CD40^+^CD83^+^CD86^+^CD11b^-^ cells [22, 23] following the same strategy as for B16F10 cells (data not shown). Moreover, melanoma infected with LM^WT^ produced high levels of the pro-inflammatory cytokines MCP-1, TNF-α, and IL-12 (Fig. 1E) as well as high production of nitric oxide, NO (Table 1), a classical listericidal reactive intermediate [18] also involved in tumour regression. We previously reported this characteristic DC phenotype and cytokine pattern on a dendritic-based vaccine for listeriosis that triggers a pro-inflammatory CD8^+^ T cell based immune response [15]. LM^WT^ infection of other APC such as J-774 macrophages does not cause transformation into DC-like phenotypes (*S1 Fig., panel C*), either production of the pro-inflammatory cytokines above-mentioned [15, 17]. In brief, LM^WT^ infection transformed melanoma into matured DC with competent APC functions and potential for tumour therapies.

**Table 1:** *Listeria* infection of melanoma induces nitric oxide and iNOS expression.

CONDITION^a^	B16F10-NI	B16F10-LM^WT^	B16F10-LM^WT^	BMDC-NI
**NO production^b^**	0.5 ± 0.02	7.5 ± 0.2	0.6 ± 0.01	8.2 ± 0.5
**APC-markers^c^**	**APC-markers**	**APC-markers**	**APC-markers**	**APC-markers**
CD11c^+^	22 ± 0.2	22 ± 0.2	92 ± 0.3	**23**
CD11b^+^	9 ± 0.1	10 ± 0.1	46 ± 0.2	13 ± 0.1
MHC-II^+^	53 ± 0.1	83 ± 0.2	65 ± 0.3	98 ± 0.3
CD86^+^	17 ± 0.2	72 ± 0.3	47 ± 0.2	87 ± 0.2
iNOS^+^	2 ± 0.1	62 ± 0.1	4 ± 0.1	66 ± 0.1

^a^B16F10 murine melanoma or BMDC were infected with LM^WT^ (B16F10-LM^WT^, BMDC-LM^WT^) or non-infected (B16F10-NI, BMDC-NI) for 24 hours.

^b^NO produced was measured in cell supernatants. Results are expressed as nmol of NO produced by 10^5^ cells (mean ± SD, *P*<0.005) obtained with triplicate samples.

^c^Cell surfaces markers of B16F10 and BMDC infected or not with LM^WT^ were analysed by FACS using the following antibodies: CD11c-PE, iNOS-FITC, CD86-V450 and MHC-II-APC. Samples were acquired using FACSCanto flow cytometer. Results are expressed as the percentages of positive cell (mean ± SD, *P*<0.005).

### *Listeria* therapy of melanoma causes tumour regression by apoptosis and bacterial clearance

**Fig 2:**
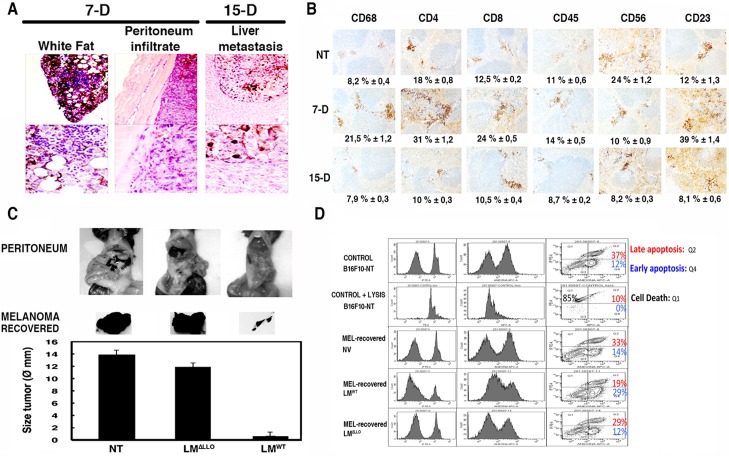
*Listeria* vaccination of melanoma shows a dual action, tumour regression by apoptosis and bacterial clearance. ***A***, C57BL/6 female were inoculated *i*.*p*. with 5 x 10^5^ B16F10/mice (n = 5) for 7 (7-D) or 15 days (15-D). Mice were bled, sacrificed and treated for histological analysis as described in *Material and Methods*. Images correspond to sections of peritoneum infiltrates or liver metastases. ***B***, C57BL/6 female were inoculated *i*.*p*. with 5 x 10^5^ B16F10/mice (n = 5) as in ***A*** for none (NT), 7 (7-D) or 15 days (15-D). Spleens from sacrificed mice (n = 5) were stained for histological analysis using different antibodies as described in *Material and Methods* and images correspond to sections. Results are expressed as percentages of positive cells (mean ± SD) (*P* < 0.05). ***C***, C57BL/6 female were inoculated *i*.*p*. with 5 x 10^5^ B16F10/mice (n = 5) for 7 days and next injected *i*.*p*. or not (NT) with 5 x 10^3^ bc/mice of different LM strains (LM^WT^ or LM^ΔLLO^) for 5 additional days. Mice were sacrificed, bled to collect sera and photographed before collecting melanoma and lungs. Images correspond to the peritoneum of mice and the recovered melanoma. Plots correspond to measurements of diameters of collected melanoma. Results are expressed as the mean ± SD (*P* < 0,05). ***D***, Melanoma recovered from LM^WT^ or LM^ΔLLO^ vaccinated mice or from non-vaccinated mice (NV) as in ***C*** were analysed for early and late apoptosis by FACS according to *Materials and Methods* after double staining with 7-AAD (IP labelled) and annexin V (anexina labelled). Results are expressed as the percentages of late apoptotic cells, necrotic death, (Q2 area corresponding to double positive for 7-AAD and annexin V cells) and the percentages of early apoptotic cells, programmed cell death (Q4 area corresponding to annexin V positive while 7-AAD negative cells) (mean ± SD) (*p* < 0.05).

To evaluate the potential of a LM^WT^ based therapy for melanoma, we set up a model of carcinomatous peritonitis induced by inoculation of B16F10 melanoma into C57BL/6 mice peritoneum for different times, 7, 15 or 23 days. The progression of melanoma in this model was as follows, after 7 days of melanoma inoculation, there is an infiltrate of tumour cells in the peritoneum (dark staining in peritoneum images of Fig. 2A) and melanoma colonization of the white fat of peritoneal organs with no infiltration on the green fat (white fat images of Fig. 2A). At 7 days, this melanoma model activated innate immune responses characterized by increased percentages of splenic CD68^+^ macrophages (from 8,2% to 21,5% values) and follicular CD23^+^ DCs (from 12% to 39% values) but decreased percentages of CD56^+^ NK cells (from 26% to 10% values) observed in histological sections. T cell responses were also stimulated with increased percentages of CD4^+^ (from 18% to 31% values) and CD8^+^ T cells (from 12,5% to 24% values) (Fig. 2B). However, the significant growth of melanoma in the peritoneum after 7 days, predicted that melanoma induced T cell responses were not tumoricidal but melanoma tolerant. In fact, after 15 days, there is an exaggerated melanoma growth in the peritoneum and metastases in lungs and livers (liver metastasis images of Fig. 2A). The immune responses become down regulated after 15 days (15-D images of Fig. 2B) and melanoma tolerance allowed tumour growth without control and the death of most of the mice after 23 days. Since bacterial vaccinations required at least 5 days for developing optimal innate and specific immune responses, we chose 7 days of melanoma induction to initiate the bacterial therapies, assuring maximal physiological conditions of the mice.

LM^WT^ therapy of melanoma using low doses of the pathogen (5 x 10^3^ CFU/mice) caused 10-fold reductions on the melanoma size after 5 days post-challenged (LM^WT^ bars in Fig. 2C). LLO is required for melanoma regression since therapies with LM^ΔLLO^ strains caused no effect on the melanoma size (LM^ΔLLO^ bars in Fig. 2C). Subcutaneous (*s*.*c*) inoculation of melanoma into new-born pups followed by LM^WT^ therapy (*s*.*c*) also reduced 6-fold the tumour size in different mice strains (*S2 Fig., panel A and B*). These results suggested that melanoma regression seemed to occur also in the skin of immune tolerant post-natal mice of different gene backgrounds. In the peritoneal carcinomatous B16F10 induced model, we also observed that reduction of the tumour size correlated with 10-fold reduction of melanoma adherence and mitotic indexes (Table 2), indicating alterations on their cell cycle due to apoptosis or necrotic death. To differentiate both types of death, we examined early and late apoptosis ratios in recovered melanoma after vaccination with LM^WT^, LM^ΔLLO^ or no vaccination (NV). Direct LM^WT^ infection of melanoma caused no change in late or early apoptosis in melanoma controls (CONTROL B16F10-NT plots of Fig. 2D). An increase in late apoptosis would correspond to necrotic death, while increases in early apoptosis ratios would indicate programmed cell death by stimuli such as cytokines. Melanoma recovered from LM^WT^ vaccinated mice showed 2-fold increases on early apoptosis, indicating that melanoma eradication occurred by programmed cell death and not by necrotic death (MEL-recovered LM^WT^ plots of Fig. 2D). Non-vaccinated mice or LM^ΔLLO^ vaccinated showed no effect in melanoma cell cycle (MEL-recovered NV and MEL-recovered LM^ΔLLO^ plots of Fig. 2D). Tumour regression also correlated with high LM^WT^ tropism for melanoma as we localized 45% of LM^WT^ vaccination in the recovered melanoma (legend in section *d* of Table 2). These results indicated that melanoma regression required LM phagocytosis by tumour cells. Therefore, we next examined the different phagocytic and APC capacities of the recovered melanoma. We observed 3-fold increases in the microbicidal abilities of the recovered melanoma after LM^WT^ vaccination since LM^WT^ phagocytic rates and replication indexes decreased (LM^WT^-MEL in Table 2). We also detected an increase in the APC competences since examination of cell surface markers verified that LM^WT^ vaccination of melanoma shifted the matured DC phenotype to a fully activated DC pattern in the recovered melanoma (LMWT-MEL under APC markers column of Table 2), namely MHC-II^+++^CD11c^++^CD8α^++^CD40^++^CD83^++^CD86^++^CD11b^+/-^F4/80^-^. These results strongly predicted elimination of the pathogen and amplification of T cell responses as melanoma transformed DC might present antigens to CD4 or CD8 T cells.

**Table 2:** *Listeria* therapy produces modification of melanoma parameters.

Condition^a^	B16F10 CONTROL	B16F10-NT	B16F10-NT	B16F10-NT
		MEL-NV	LM^WT^-MEL	LM^ΔLLO^-MEL
**Adherence** ^**b**^	100 ± 2	100 ± 2	5 ± 0.2	95 ± 0.2
**Mitotix index** ^**c**^	2 ± 0.01	2 ± 0.03	0.1 ± 0.02	1.9 ± 0.02
^**d**^ **Phago-R-LM** ^**WT**^	190 ± 11	192 ± 12	60 ± 3	180 ± 3
**Phago-R-LM** ^**ΔLLO**^	185 ± 9	185 ± 11	61 ± 2	188 ± 2
^**e**^ **RI-LM** ^**WT**^	70 ± 2	72 ± 0.32	12 ± 0.02	67 ± 0.2
**RI-LM** ^**ΔLLO**^	0.5± 0.01	0.5± 0.01	0.10± 0.01	0.6± 0.01
**APC-markers** ^**f**^	**APC-markers**	**APC-markers**	**APC-markers**	**APC-markers**
**CD11c** ^**+**^	25 ± 0.1	22 ± 0.1	62 ± 0.2	23
**CD11b** ^**+**^	10 ± 0.1	10 ± 0.1	6 ± 0.1	10 ± 0.1
**MHC-II** ^**+**^	53 ± 0.1	53 ± 0.2	93 ± 0.3	53 ± 0.2
**CD40** ^**+**^	25 ± 0.1	5 ± 0.1	58 ± 0.1	5 ± 0.1
**CD83** ^**+**^	18 ± 0.1	8 ± 0.1	52 ± 0.1	8 ± 0.1
**CD86** ^**+**^	17 ± 0.1	7 ± 0.1	47 ± 0.1	7 ± 0.1
**CD8alfa** ^**+**^	0.3 ± 0.01	0.5 ± 0.01	72 ± 0.2	0.7 ± 0.01
**F4/80** ^**+**^	7 ± 0.1	2 ± 0.1	1 ± 0.01	2 ± 0.1

^a^B16F10 murine melanoma (B16F10-NT) were inoculated for 7 days into mice (n = 5). Mice were next vaccinated with LM^WT^ (LM^WT^-MEL) or LM^ΔLLO^ (LM^ΔLLO^-MEL) for 5 days or non-vaccinated (MEL-NV) as described in *Material and Methods*. In some experiments we also used GFP-LM^WT^ to follow LM localization. Next, mice were sacrified and melanoma recovered from peritoneum (MEL). Melanoma size (Ø) was measured with a calliper and cells disaggregated, passed through a filter and cultured for 7 days before analysis (*P* < 0.05).

^b^Adherence was evaluated as the percentage of culture cells adhered to the culture plates after 16 hours of culture. Results are expressed as the percentage of cells (mean ± SD) (*P* < 0.05).

^c^Mitotic index was calculated as the ratio of the number of cells set in culture at time 0 hours compared to time 16 hours (mean ± SD) (*p* < 0.05).

^d^Recovered melanoma were infected with LM^WT^ or LM^ΔLLO^ for different times (0, 6, 16h) and phagocytic rates (Phago-R) were calculated as the number of CFU at 0 h. Results are expresses as CFU x 100 (mean ± SD) (*p* < 0.05)

^e^Replication indexes (RI) analysis of recovered melanoma as in d. RIs were calculated as the ratio of the number of CFU at 16 h divided by the amount of CFU at 0 h. This parameter was considered as an indicator of bacterial growth. Results are expressed as CFU (mean ± SD) (*p* < 0.05).

^f^APC markers analysed in the recovered melanoma vaccinated or not (MEL-NV) by FACS using the following antibodies: CD11c-PE, CD11b-FITC, F4/80-PE, CD40-PE, CD83-FITC, CD86-V450, anti-IA^b^-APC and CD8α-V450. Samples were acquired using FACSCanto flow cytometer and percentages of positive cells for each antibody are shown. We also vaccinated with GFP-LM^WT^ and observed that 45% of GFP-LM^WT^ bacteria were localized in the recovered melanoma (LM^WT^-MEL). Results are expressed as percentages of positive cells (mean ± SD of triplicates, *P*<0.005).

### *Listeria* vaccination of melanoma generates LLO specific CD8^+^ and CD4^+^ T cells using recombinant LLO or peptides

**Fig 3:**
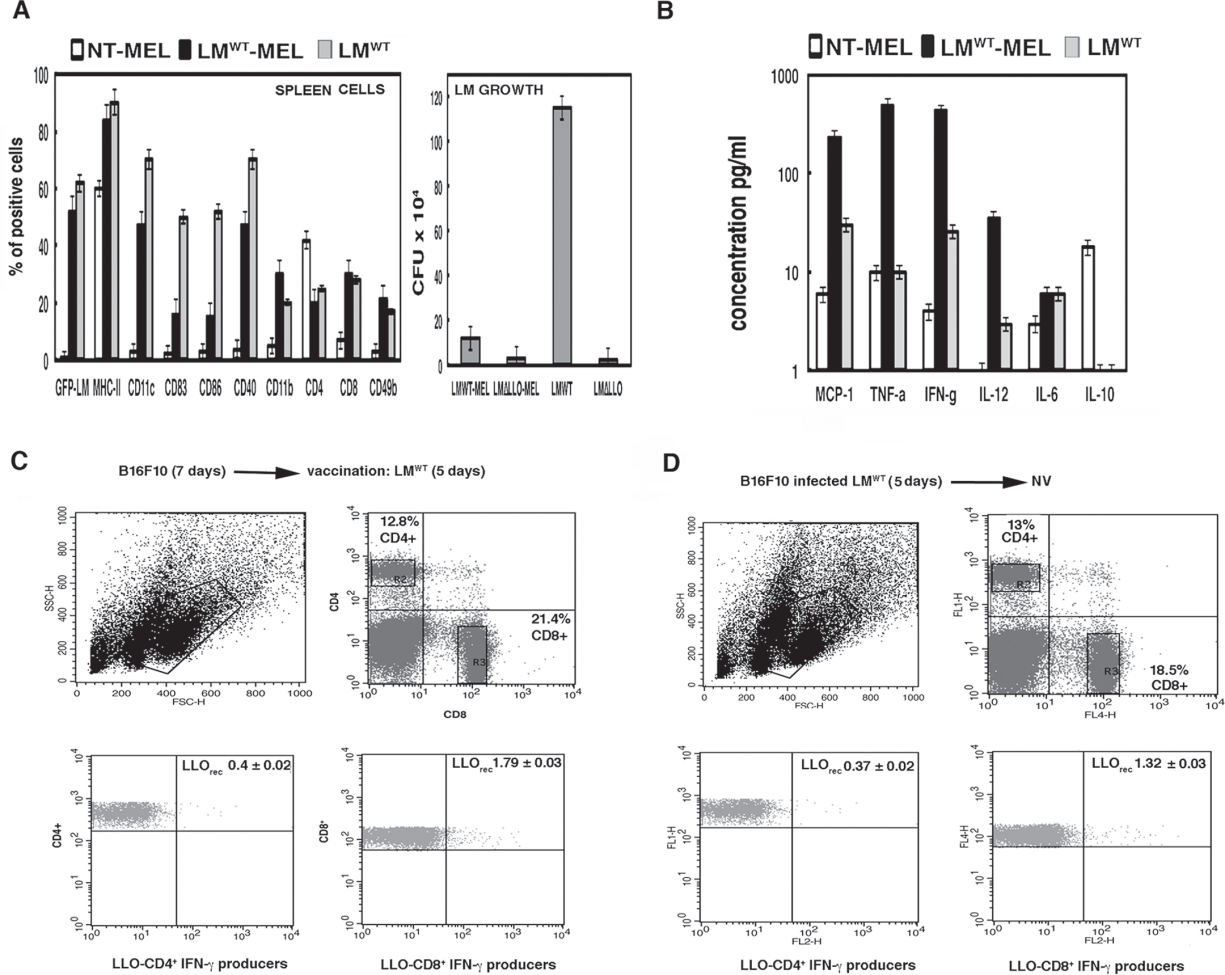
Efficiency of *Listeria* vaccination of melanoma is mediated by activation of LLO_rec_ specific CD8^+^ T cells and inhibition of LLO_rec_ specific CD4^+^ T cells. C57BL/6 female were inoculated *i*.*p*. with 5 x 10^5^ B16F10/mice (n = 5) for 7 days and next injected *i*.*p*. or not (NT) with 5 x 10^3^ bc/mice of GFP-LM^WT^ strain for 3 additional days. Mice were bled and sacrificed. ***A***, Immune cells plot (left) corresponds with spleens were homogenized and cell populations were analysed by FACS. Results were expressed as the mean of the percentages of positive cells ± SD. LM growth plot (right) corresponds with spleen homogenates examined for CFU in blood-agar plates. Results are expressed as CFU (mean ± SD) obtained with triplicate samples from three independent experiments (*P*< 0,05). ***B***, Levels of pro-inflammatory cytokines (MCP-1, TNF-alfa, IFN-gamma, IL-6, IL-10, IL-12) were analysed in sera of mice using the CBA kit (Becton Dickinson) by flow cytometry. Results were expressed as cytokine concentration (pg/ml of mean ± SD, *P*<0,05). ***C***, Spleen cells obtained from homogenates after inoculation with melanoma B16F10 (5 x 10^5^ cells/mice) for 7 days and vaccination with LM^WT^ for 5 days (LM^WT^-MEL). Cells were stimulated 5 h with recombinant LLO (0.1 μg/ml) in the presence of brefeldin A for intracellular cytokine staining. LLO-stimulated spleen cell surface was stained for CD4 or CD8 and fixed and permeabilized using cytofix/cytoperm kit. Stimulated cells were surface stained for CD4 or CD8 using anti-CD4^+^FITC-labeled or anti-CD8^+^APC-labelled and data gated to include histograms show the percentages of LLO-CD4^+^ and IFN-gamma producers (lower left) and LLO-CD8^+^ and IFN-gamma producers (lower right) (R2 and R3 gates). Experiments were performed in triplicate and results are expressed as the mean ± SD (*p* < 0.05). ***D***, Spleen cells obtained from homogenates after inoculation with melanoma B16F10 pre-infected with LM^WT^ (5 x 10^5^ cells/mice) for 7 days. Cells were stimulated 5 h with recombinant LLO (0.1 μg/ml) in the presence of brefeldin A for intracellular cytokine staining. Procedures were performed as in ***C*** and results expressed as the mean ± SD (*p* < 0.05).

To explore the immune responses generated by melanoma therapy with LM^WT^, we examined the cell populations in the spleens of mice with LM^WT^ therapy (black bars in Fig. 3A). First, we observed that 96% of the inoculated LM^WT^ localized in MHC-II^+^ cells, 56% CD11c^+^ DCs and 40% CD11b^+^ macrophages (GFP-LM black bars in Fig. 3A). These percentages were similar to the cell populations observed in classical LM^WT^ infections (GFP-LM grey bars in Fig. 3A). These results confirmed LM^WT^ tropism for APC. Second, melanoma therapy with LM^WT^ increased significantly the innate responses in the spleens since we observed amplifications on the percentages of activated DCs, MHC-II^+^CD11c^+^CD40^+^CD83^+^CD86^+^ positive cells, CD11b^+^F4/80^+^ positive macrophages (data not shown) and NK cells of the tumorigenic CD49b^+^ phenotype (black bars of Fig. 3A in left graphic). Third, we detected 200-fold increases in the levels of MCP-1, TNF-alfa, IFN-gamma and IL-12, cytokines produced after melanoma therapy with LM^WT^ (black bars in Fig. 3B) compared to non-treated mice (white bars in Fig. 3B). Melanoma therapy with LM^WT^ also altered T cell responses, increasing the percentages of CD8^+^ T cells and decreasing in the percentages of CD4^+^ T cells (CD4 and CD8 black bars on left plot of Fig. 3A). The percentages of CD4 and CD8 T cells detected after melanoma therapy with LM^WT^ were similar to the percentages observed after a classical LM^WT^ infection, suggesting that T cells were *Listeria* driven (Fig. 3A). We next examined LLO specific T cell responses induced by melanoma therapy with LM^WT^ since LLO seemed relevant in this melanoma model and in most reported *Listeria*-based immune-therapies [4, 5, 15, 24]. Therapy of melanoma with LM^WT^ stimulated higher 1.79 ± 0.03 percentages of recombinant LLO (LLO_rec_)-specific CD8^+^ cells and IFN-gamma producers and lower 0.4 ± 0.01 percentages of LLO_rec_-specific CD4^+^ cells and IFN-gamma producers (Fig. 3C). While a classical LM infection after 5 days produced similar percentages of LLO_190–201_ specific CD4^+^ and LLO_296–304_ specific CD8^+^ T cell subsets and IFN-gamma producers, 1.05 ± 0.02 and 1.02 ± 0.01 percentages, respectively (*S3 Fig., panel A*). Increased cytotoxic T cell responses seemed to explain the accelerate LM^WT^ clearance in the spleens (LM growth plot in Fig. 3A). These results strongly suggested that *in vivo* effects of melanoma therapy with LM^WT^ implies the transformation of melanoma into DC-like cells that stimulate LLO-specific T cell immune responses and caused melanoma regression. To confirm this hypothesis we first infected *in vitro* melanoma with LM^WT^ and next *i*.*p* inoculated mice with these melanoma-infected cells. Five days post-inoculation, we examined recombinant LLO_rec_-specific CD4^+^ and CD8^+^ T cells producing IFN-gamma. Similar to melanoma therapy with LM^WT^, we observed low 0.37 ± 0.02 percentages of LLO_rec_-specific CD4^+^ T cells and expanded 1.32 ± 0.03 percentages of LLO_rec_-specific CD8^+^ T cells producing IFN-gamma (Fig. 3C). We also observed decreased 0.03 ± 0.01 percentages of LLO_190–201_ specific CD4^+^ and enhanced 1.76 ± 0.03 percentages of LLO_296–304_ specific CD8^+^ T cells producing IFN-gamma (*S3 Fig., panel B*). We also evaluated immune responses to melanoma antigens using a B16F10 extract (MEL_ext_). Control melanoma inoculation produced 0.60 ± 0.02 percentages of MEL_ext_-specific CD4^+^ and 0.65 ± 0.03 percentages of MEL_ext_-specific CD8^+^ T cells producing IFN-gamma (*legend of S3 Fig., panel C*). Pre-infection of melanoma with LM^WT^ and inoculation into mice presented diminished 0.15 ± 0.01 percentages of MEL_ext_-specific CD4^+^, while no alteration of 0.65 ± 0.02 percentages of MEL_ext_-specific CD8^+^ T cells (*S3 Fig., panel C*). These results suggested that LM^WT^ therapy induces LLO, but not melanoma-specific CD8^+^ T cell expansion; while decreases both LLO and melanoma-specific CD4^+^ T cells. We confirmed the LLO specific CD8^+^ T cell expansion examining the frequencies of LLO_296–304_ specific CD8^+^ T cells (Table 3). Melanoma therapies with LM^WT^ increased LLO_296–304_ frequencies, from 1.75 values of a classical LM^WT^ infection to 2.25–2.50 values (B16F10→LM^WT^ and B16F10-LM^WT^→NV rows in Table 3). The increase in LLO specific CD8^+^ T cell responses, correlated with regression of melanoma size observed in mice inoculated with melanoma pre-infected with LM^WT^ or control melanoma (*S3 Fig., panel D*). This is a widely common mechanism of several pathogenic bacteria used in cancer therapies [1, 4, 25]. In fact, other *Listeria*-based vaccines against breast cancer function by similar mechanisms [5].

**Table 3:** Frequencies of LLO_296–304_ specific CD8^+^ T cells in spleens of B16F10 treated mice or non-treated after LM^WT^ therapy.

Therapy condition	% Total dimer-CD8/LLO_296–304_	% Gated dimer-CD8/LLO_296–304_
**NT** ^**a**^ **→ LM** ^**WT**^	0.09 ± 0.01	1.75 ± 0.01
**B16F10** ^**b**^ **→ LM** ^**WT**^	0.08 ± 0.02	2.53 ± 0.05
**B16F10** ^**c**^ **-LM** ^**WT**^ **→NV**	0.06 ± 0.01	2.25 ± 0.01

^a^C57BL/6 mice non-treated with murine melanoma were inoculated with saline *i*.*p* for 7 days and next injected *i*.*p* with LM^WT^ for 3 days as described in *Materials and Methods*. Splenocytes from mice treated with LM^WT^ were incubated with recombinant dimeric H-2K^b^: Ig fusion protein (BD Biosciences) loaded with LLO_296–304_ peptide. The staining cocktail contained the dimeric fusion protein loaded with the peptides, CD8 and anti-IFN-gamma antibodies. CD8^+^ cells were gated for anti-IFN-gamma staining (% Gated dimer-CD8) to calculate the frequencies of CD8^+^-LLO_296–304_. Results are expressed as percentages of triplicate samples ± SD. *P*<0.05.

^b^B16F10 murine melanoma were inoculated *i*.*p* into C57BL/6 mice for 7 days and next injected *i*.*p* with LM^WT^ for 3 additional days as described in *Materials and Methods*. Splenocytes from mice were incubated with recombinant dimeric H-2Kb: Ig fusion protein as in a. *P*<0.05.

^c^B16F10 murine melanoma pre-infected with LM^WT^ was inoculated into C57BL/6 mice for 7 days as described in Materials and Methods. Splenocytes from mice were incubated with recombinant dimeric H-2Kb: Ig fusion protein as in a. *P*<0.05.

**Fig 4:**
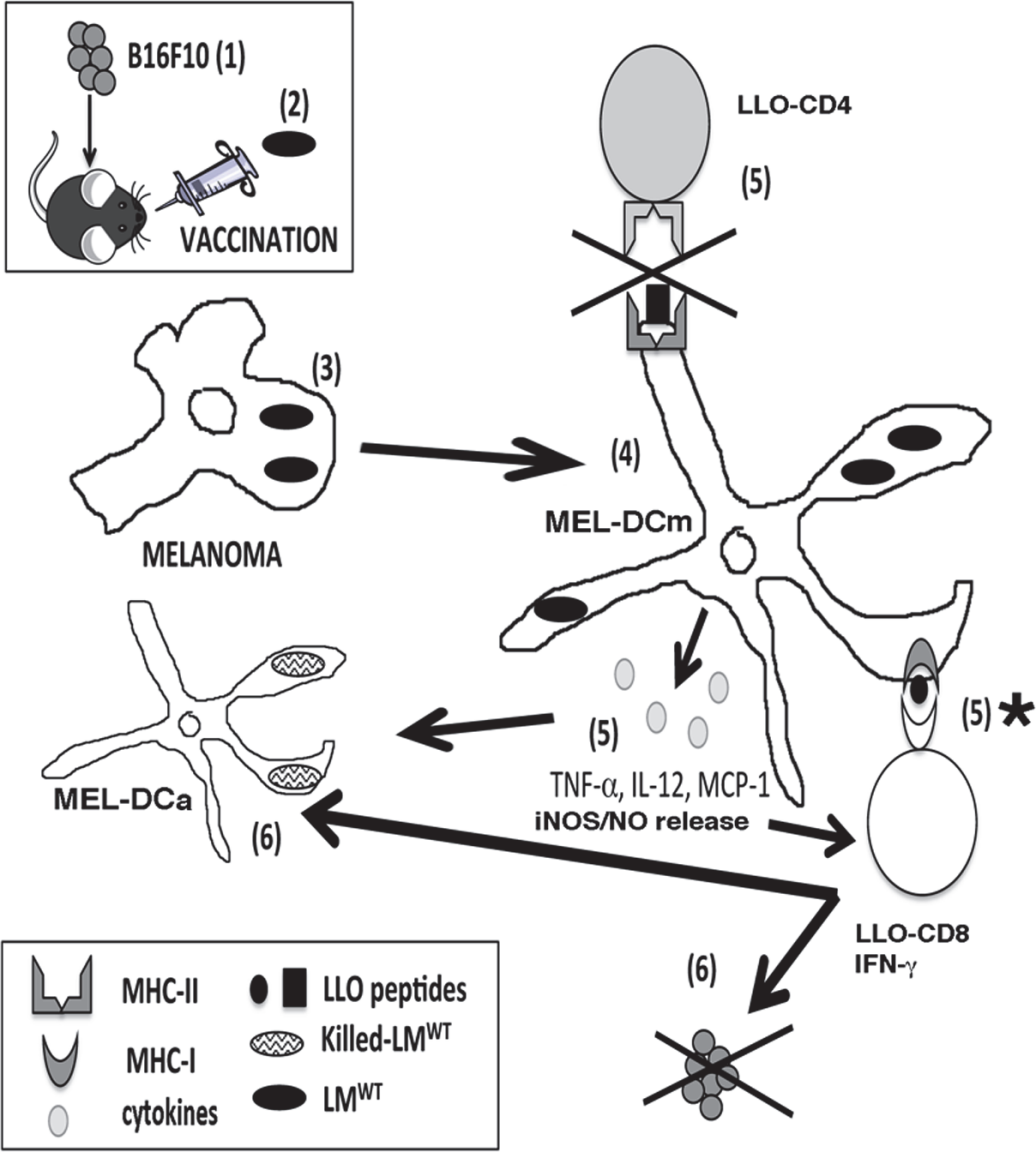
Model of action of our LM^WT^ vaccination of melanoma. After LM^WT^ vaccination of melanoma induce mice, bacteria would infect the melanoma and transformed them into matured dendritic-like cells (MEL-DCm) (*step 4*) with APC competence to process LLO and generate LLO_189–201_/CD4^+^-restricted and LLO_91–99_/CD8^+^ restricted peptides. Melanoma transformation into DCm generates exaggerated levels of the pro-inflammatory cytokines/chemokines, MCP-1, TNF-alfa and IL-12 (*step 5*), amplifies the activation of LLO_91–99_/CD8^+^-restricted T cells (*step 5*) and blocks the stimulation of LLO_189–201_/CD4^+^-restricted T cells (*step 5*). The exaggerated levels of pro-inflammatory cytokines and the amplification of LLO_91–99_/CD8^+^-restricted T cells producing high levels of IFN-gamma, strongly activated innate cells with tumoricidal potential. The amplification of LLO_91–99_/CD8^+^-restricted T cells would cause a LLO-driven tumour destruction by programmed cell death (*step 6*). Also these LLO_91–99_/CD8^+^-restricted T cells producing high levels of IFN-gamma would activate the LM^WT^ infected melanoma (MEL-Dm) to a fully activated state (MEL-DCma) that might eliminate the pathogen (*step 6*) with the advantage of not requiring antibiotic treatment to destroy this low dose of the pathogen used as therapeutic vaccine.

In conclusion, we envision the following model (Fig. 4) to explain the efficiency of our LM^WT^ vaccination. After the melanoma model is established in the mice for 7 days (*step 1*), vaccination with low doses of pathogenic LM^WT^ for 5 days (*step 2*) accessed the growing tumour and infected these cells (*step 3*). LM^WT^ infection of melanoma transformed them into matured dendritic-like cells (MEL-DCm) (*step 4*) with APC competence and MIIC phagosomes that degraded LLO to LLO processed forms LLO_1–491_ that allowed the generation of LLO restricted CD4^+^ T cells and LLO restricted CD8 T cells. This *Listeria*-driven melanoma transformation also generates exaggerated levels of the pro-inflammatory cytokines/chemokines, MCP-1, TNF-α and IL-12 and high production of nitric oxide (NO), a well known listericidal and tumour reducer molecule (*step 5*) [18]. This LM-driven melanoma transformation also amplifies the activation of LLO_296–304_ restricted CD8^+^ T cells (*step 5*) and blocks the stimulation of LLO_190–201_ restricted CD4^+^ T cells (*step 5*) [28]. The exaggerated levels of pro-inflammatory cytokines and NO combined with the amplification of LLO_296–304_-restricted CD8^+^ T cells producing high levels of IFN-gamma, strongly activated innate cells with tumoricidal potential such as CD11b^+^F4/80^+^ macrophages and specially, the NK tumoricidal phenotype cells CD49b^+^ and also other activated DC. But also, this dual immune action unbalanced LLO-specific immune response towards a prevalence of LLO-specific CD8^+^ T cell subsets with reduction of the LLO-specific CD4^+^ T cell subsets (*step 5*), as well as a general reduction on CD4 T cell responses. This reduction on CD4 T cells, *Listeria*-specific or not, might break tumour tolerance and in combination with the cytotoxic phenotypes of NK CD49b^+^ and LLO-restricted CD8^+^ T cells [5, 6, 15, 17], would cause a LLO-driven tumour destruction by programmed cell death (*step 6*). Also these LLO-restricted CD8^+^ T cells producing high levels of IFN-γ would activate the LM^WT^ infected melanoma (MEL-Dm) to a fully activated state (MEL-DCma), with a TipDC phenotype of CD11c^++^MHC-II^++^CD8α^++^CD40^++^CD86^++^F4/80^-^ reported by several groups [29, 30], that might eliminate the pathogen (*step 6*) with the advantage of not requiring antibiotic treatment to destroy this low dose of the pathogen used as therapeutic vaccine.

The success of our LM^WT^ vaccination of melanoma seemed exerted by matured DC or melanoma transformed into DC (MEL-DCm or MEL-DCma) that strongly stimulate LLO-restricted CD8^+^ T cells. This mechanism of action resembles the efficiency of a recent dendritic-based vaccination against listeriosis that our group, as well as others [15, 26], reported using DC loaded with LLO_91–99_ peptide and open up future dendritic-based vaccination protocols for melanoma using *Listeria* peptides.

The carcinomatous peritonitis model of melanoma used in this study resembles at day 7, stages II and III of human melanoma categories established in 2009 by the American Joint Committee on Cancer [27] that corresponded with any size of tumour and no propagation to other organs. Therefore, we propose that our LM^WT^ vaccination of melanoma with low doses of pathogenic bacteria might be a safe therapy for stages II and III of human melanoma, where the only efficient treatment is currently surgery. Pre-clinical studies with melanoma patients currently analyse this hypothesis (SYD and CAD unpublished results). In fact, we predicted that LM^WT^ vaccinations of cutaneous melanoma might transform melanoma cells into mature and activated DCs and stimulate tumoricidal *Listeria*-specific CD8^+^ T cells and CD49b^+^ NK cells as well as generate IFN-gamma and IL-12 cytokines effective against tumours as well as killing the pathogen without topical antibiotic treatment. Here we proposed that targeting of *Listeria* to melanoma and transforming melanoma into dendritic cells is a new mechanism of vaccine efficacy that can be applied to attenuated *Listeria* vaccines expressing tumour antigens proposed currently as therapies [19, 31].

## Supporting Information

S1 Fig *Listeria* does not transform other APC and non-APC cells into a dendritic cell phenotype. ***A***, Kinetic analysis of J-774 macrophages (APC) and non-APC murine embryonic fibroblasts (MEF) or the ovarian CHO tumour cell lines infected with different LM strains (LM^WT^, LM^ΔLLO^). Results are expressed as CFU (mean ± SD) obtained with triplicate samples from three independent experiments (*P*<0.05). ***B***, Western blots of 30 μg of purified phagosomes from J-774, BMDC, B16F10 melanoma for different MIIC markers: a/b stable MHC-II chains; Rab5a and LLO_1–491_ forms bound to MHC-class II molecules. ***C***, J-774 macrophages infected with LM strains or non-infected (NI) were surface stained for the following markers: CD11c-PE, CD11b-FITC, F4/80-PE, CD40-PE, Gr-1-FITC and anti-IA^b^-APC. Samples were acquired using FACSCanto flow cytometer and percentages of positive cells for each antibody are shown. Results are expressed as the mean ± SD of triplicates (*p*<0.05).
(TIF)

S2 Fig Vaccination of neonates with *Listeria* causes regression of subcutaneous melanoma. 2 days post-natal CD-1 (black bars) or C57BL/6 (grey bars) neonates were inoculated *s*.*c* with 1 x 10^5^ B16F10/mice (n = 10) for 7 days and next injected *s*.*c* or not (NT) with 1 x 10^3^ bc/mice of different LM strains (LM^WT^ or LM^ΔLLO^) for 5 additional days. Mice were sacrificed, photographed before collecting melanoma and melanoma weighted and sized with a calliper. *Panel A*, shows plots that correspond to measurements of diameters of collected melanoma. Results are expressed as the mean ± SD (*P* < 0,05). *Panel B*, shows images of control melanoma (MEL-NT) or melanoma vaccinated with LM^WT^ (MEL-WT) inoculated *s*.*c* into C57BL/6 mice.
(TIF)

S3 Fig Specific LLO immune responses in melanoma therapies with LM and tumour regression. LLO specific immune responses were examined in splenocytes with LLO_190–201_ and LLO_296–304_ peptides specific for CD4 or CD8 T cells in C57BL/6 mice [27], respectively, by intracellular cytokine staining. Mice were injected with LM^WT^ for 5 days without melanoma challenge (*panel A*) or inoculated with melanoma pre-infected with LM^WT^ (*panels B and C*). *Panel A*, shows the percentages of LLO_190–201_ specific CD4 or LLO_296–304_ specific CD8 T cells mice infected with LM^WT^ for 5 days. *Panel B*, shows the percentages of LLO_190–201_ specific CD4 or LLO_296–304_ specific CD8 T cells in mice inoculated with melanoma pre-infected with LM^WT^. *Panel C*, shows specific melanoma immune response using a B16F10 extract (MELext) and examining the percentages of MEL_ext_-specific CD4 or CD8 T cells of experiment of *panel B* by intracellular cytokine staining. Inoculation of control melanoma showed 0.60 ± 0.01 percentages of MEL_ext_-specific CD4 T cells and 0.65 ± 0.01 percentages of MEL_ext_-specific CD8 T cells. Panel D, shows the melanoma size of experiment of *panel C*, melanoma pre-infected with LM^WT^ (B16F10-LM-WT bars) or control melanoma (B16F10 bars). Results are expressed as the mean ± SD. *P*<0.05.
(TIF)
