# Supplementary figures and images for: A Novel Therapy for Melanoma Developed in Mice: Transformation of Melanoma into Dendritic Cells with Listeria monocytogenes
Source: PLoS One. 2015 Mar 11;10(3):e0117923. doi: 10.1371/journal.pone.0117923 (PMC4356589; doi:10.1371/journal.pone.0117923)

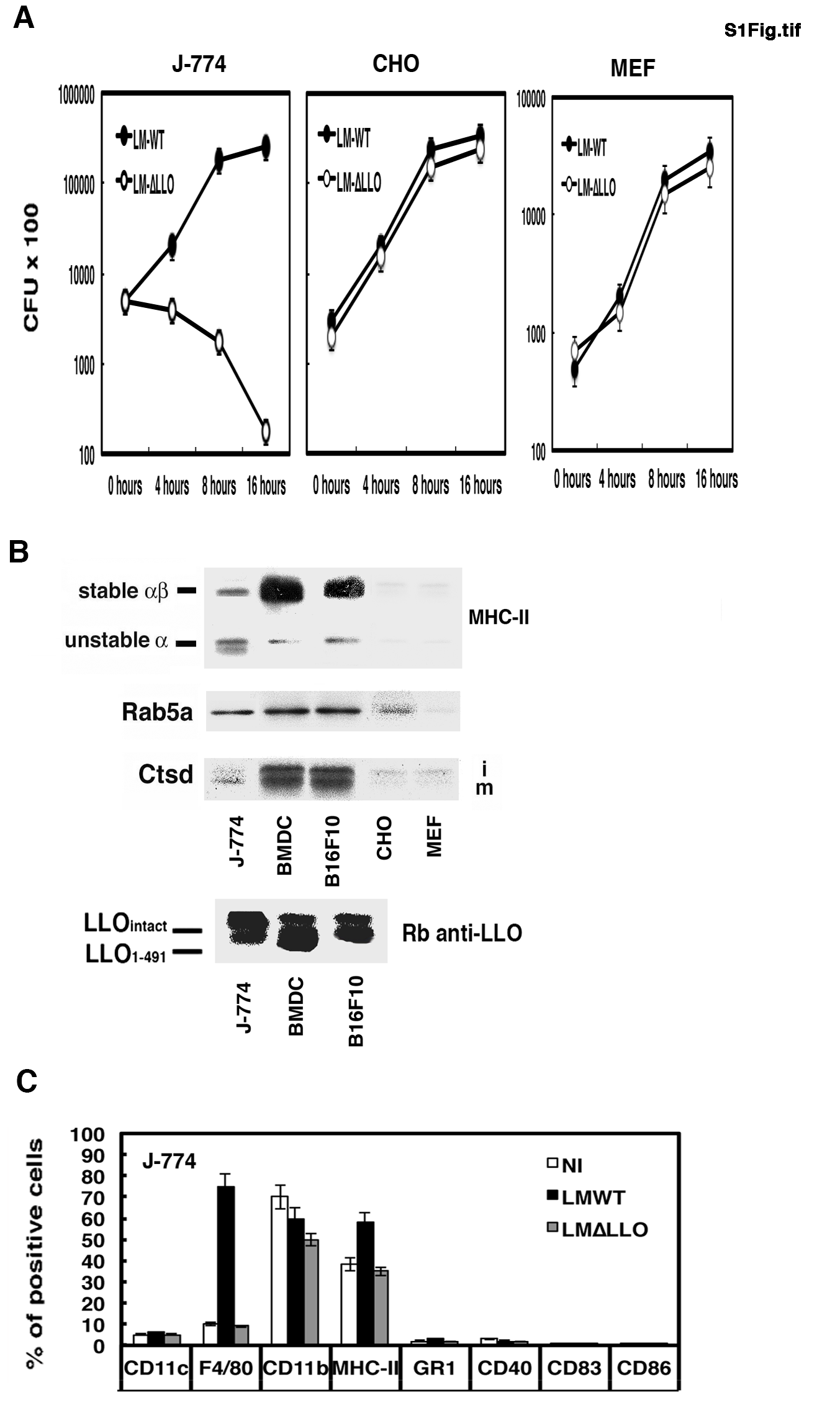

Supplement: S1 Fig — A, Kinetic analysis of J-774 macrophages (APC) and non-APC murine embryonic fibroblasts (MEF) or the ovarian CHO tumour cell lines infected with different LM strains (LMWT, LMΔLLO). Results are expressed as CFU (mean ± SD) obtained with triplicate samples from three independent experiments (P<0.05). B, Western blots of 30 μg of purified phagosomes from J-774, BMDC, B16F10 melanoma for different MIIC markers: a/b stable MHC-II chains; Rab5a and LLO1–491 forms bound to MHC-class II molecules. C, J-774 macrophages infected with LM strains or non-infected (NI) were surface stained for the following markers: CD11c-PE, CD11b-FITC, F4/80-PE, CD40-PE, Gr-1-FITC and anti-IAb-APC. Samples were acquired using FACSCanto flow cytometer and percentages of positive cells for each antibody are shown. Results are expressed as the mean ± SD of triplicates (p<0.05). (TIF) [file pone.0117923.s001.tif]

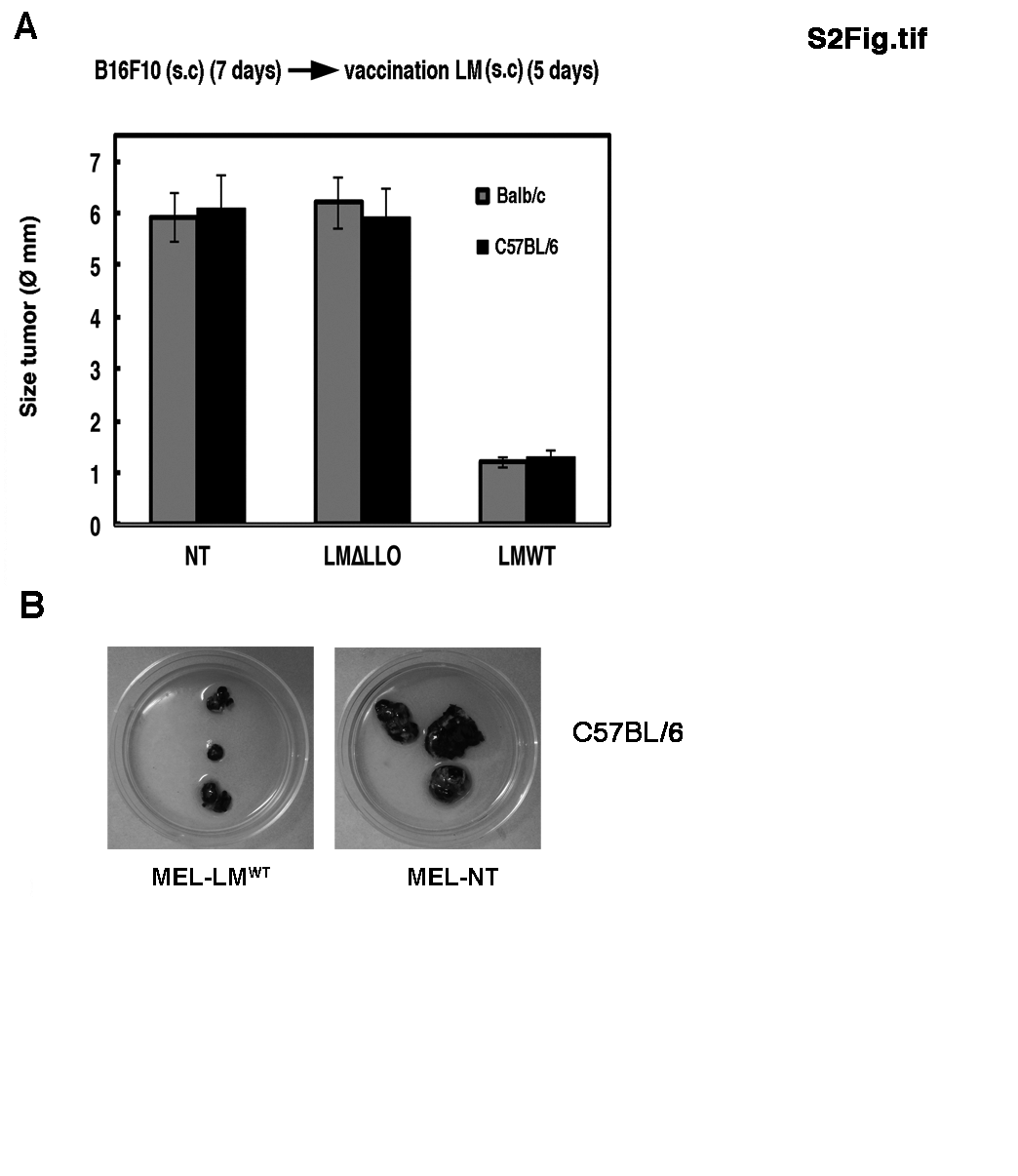

Supplement: S2 Fig — 2 days post-natal CD-1 (black bars) or C57BL/6 (grey bars) neonates were inoculated s.c with 1 x 10^5^ B16F10/mice (n = 10) for 7 days and next injected s.c or not (NT) with 1 x 10^3^ bc/mice of different LM strains (LMWT or LMΔLLO) for 5 additional days. Mice were sacrificed, photographed before collecting melanoma and melanoma weighted and sized with a calliper. Panel A, shows plots that correspond to measurements of diameters of collected melanoma. Results are expressed as the mean ± SD (P < 0,05). Panel B, shows images of control melanoma (MEL-NT) or melanoma vaccinated with LMWT (MEL-WT) inoculated s.c into C57BL/6 mice. (TIF) [file pone.0117923.s002.tif]

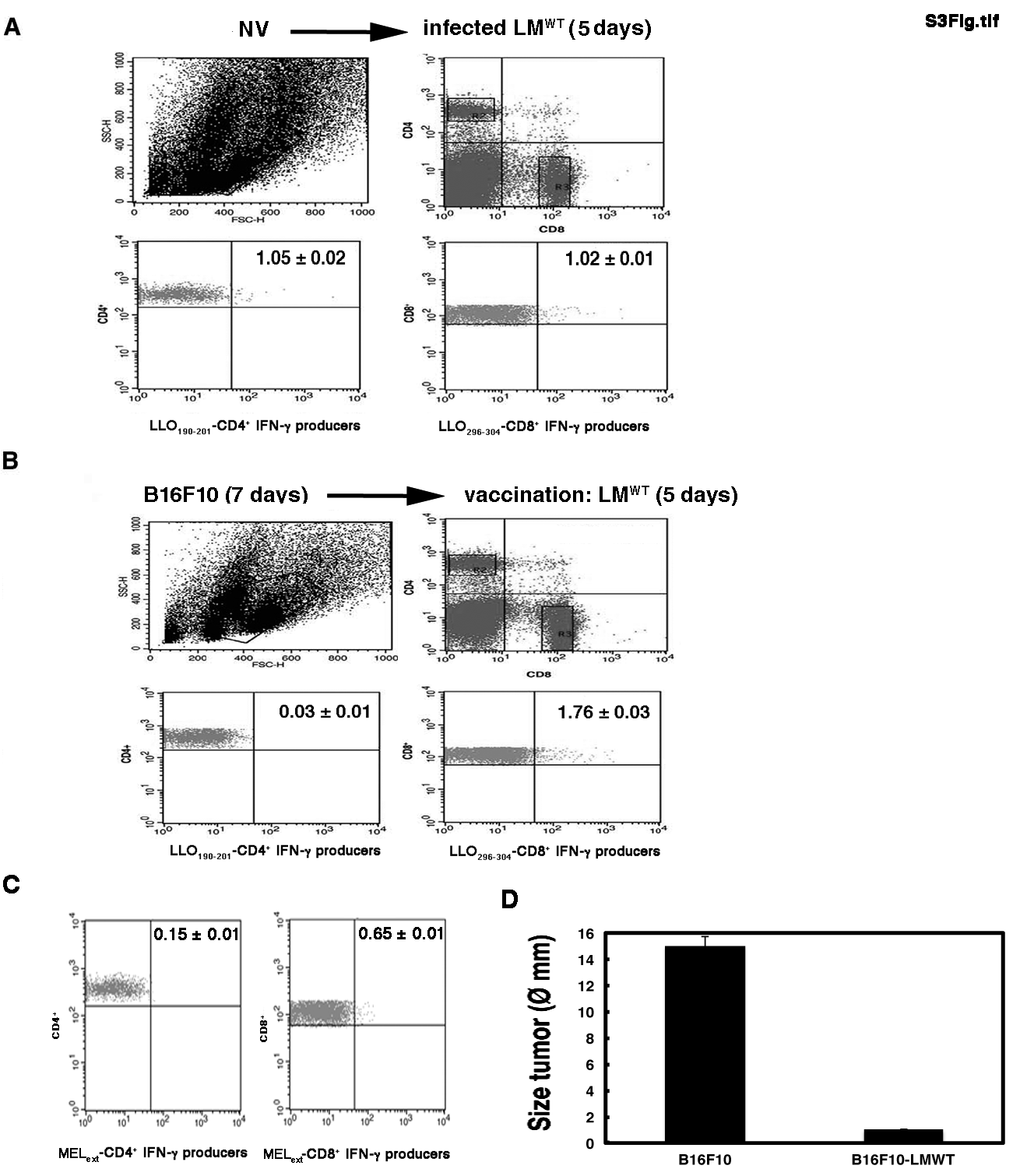

Supplement: S3 Fig — LLO specific immune responses were examined in splenocytes with LLO190–201 and LLO296–304 peptides specific for CD4 or CD8 T cells in C57BL/6 mice [27], respectively, by intracellular cytokine staining. Mice were injected with LMWT for 5 days without melanoma challenge (panel A) or inoculated with melanoma pre-infected with LMWT (panels B and C). Panel A, shows the percentages of LLO190–201 specific CD4 or LLO296–304 specific CD8 T cells mice infected with LMWT for 5 days. Panel B, shows the percentages of LLO190–201 specific CD4 or LLO296–304 specific CD8 T cells in mice inoculated with melanoma pre-infected with LMWT. Panel C, shows specific melanoma immune response using a B16F10 extract (MELext) and examining the percentages of MELext-specific CD4 or CD8 T cells of experiment of panel B by intracellular cytokine staining. Inoculation of control melanoma showed 0.60 ± 0.01 percentages of MELext-specific CD4 T cells and 0.65 ± 0.01 percentages of MELext-specific CD8 T cells. Panel D, shows the melanoma size of experiment of panel C, melanoma pre-infected with LMWT (B16F10-LM-WT bars) or control melanoma (B16F10 bars). Results are expressed as the mean ± SD. P<0.05. (TIF) [file pone.0117923.s003.tif]
